# Age- and oxidative stress-induced centrosome amplification and renal stones in *Drosophila* Malpighian tubules

**DOI:** 10.1242/bio.061743

**Published:** 2024-12-16

**Authors:** Hyun-Jin Na, Mi-Jeong Sung, Joung-Sun Park

**Affiliations:** ^1^ Aging and Metabolism Research Group, Division of Food Functionality Research, Korea Food Research Institute, Wanju 55365, Republic of Korea; ^2^ Institute of Nanobio Convergence, Pusan National University, Busan 46241, Republic of Korea; ^3^ Department of Molecular Biology, Pusan National University, Busan 46241, Republic of Korea

**Keywords:** *Drosophila*, Aging, Malpighian tubules, Centrosome amplification, Renal stone, Aging marker

## Abstract

Renal diseases, including cancer, are rapidly increasing worldwide, driven by rising temperatures and changing diets, especially among younger people. Renal stones, a major risk for chronic renal disease, are increasingly common due to various health issues. Research on the underlying mechanisms, drug discovery, and the effects of aging and stress is limited. We used *Drosophila*, due to its similarity to the human renal system and ease of use, to identify cancer hallmarks and renal stone formation related to aging and oxidative stress. Our results indicate that centrosome amplification and stone formation increase with age and oxidative stress, and high sucrose feeding also heightens stone formation in the renal system. Our results show a close relationship between these diseases and aging, reactive oxygen species (ROS) stress, and chronic diseases. We suggest that the *Drosophila* renal model could be a powerful tool to study the relationship between age and age-related diseases and to discovering new agents for nephropathy.

## INTRODUCTION

Aging is intricately influenced by cellular damage, genetic changes, and environmental factors, making it a risk factor for a multitude of age-related diseases including cancers and type 2 diabetes (Guo et al., 2022). Age-related deterioration makes older individuals more susceptible to renal diseases, such as chronic renal disease and acute renal injury (Yuan et al., 2022). Therefore, it is important to study the relationships between aging, reactive oxygen species (ROS) stress, and renal-related diseases, including renal cancer and stones.

Renal stones are one of the major risk factors for chronic renal-related diseases, and their prevalence has increased with changes in eating habits and increases in various human conditions, such as obesity, diabetes, and aging (Alelign and Petros, 2018; Chou et al., 2011). Renal stones are a global problem that affects approximately 12% of the global population at least once in our lifetime (Alelign and Petros, 2018). Research on renal stones is needed to identify palliative agents and treatments for renal stone disease and to understand the relationship between renal stone disease and various genetic, environmental, and metabolic factors, such as obesity and diabetes (Shen et al., 2023; Xu et al., 2023). Several animal model system studies have been conducted for renal stone research, including rat, mouse, pig, and dog models (Khan, 1997). In particular, rat models rely on dietary or intraperitoneal injections of lithogenic agents such as ethylene glycol and ammonium chloride to induce calculus formation (Khan, 1997; Ali et al., 2018). However, stone formation is inconsistent, producing variable results in this model system. Therefore, new animal models are required to study stone formation, and may provide better results in stone disease research, revealing the root causes and new treatment approaches.

*Drosophila* is an excellent model system for studying various human diseases, including metabolism, aging, and cancer (Herranz and Cohen, 2017). Its rapid reproductive rate, largely understood genome, and experimental simplicity make *Drosophila* a potent model system (Ali et al., 2018). *Drosophila* organs are functionally and structurally well preserved, and tools for their genetic regulation as tissue- and cell-type-specific lines are available for a variety of studies (Mirzoyan et al., 2019). The renal of *Drosophila* consists of two components: renal cells and Malpighian tubules (MTs). The MTs of *Drosophila* are similar to mammalian renal tubules both functionally and structurally (Wang et al., 2022), including the active transport of water, ions, and solutes into the MT lumen to produce urine (Cohen et al., 2020). In recent studies, the *Drosophila* renal system has been used as a research model for human renal cancer (Dow and Romero, 2010). Although cell proliferation assessments, such as clone analysis and EdU assay, are well established in the *Drosophila* renal system (Wang and Spradling, 2020; Kwok et al., 2024 preprint), direct hallmarks for renal cancer are urgently needed. Previously, we reported that centrosome amplification (CA) in mitotic intestinal stem cells (ISCs) is a useful marker for aging stem cells and cancer in the *Drosophila* gut (Park et al., 2014, 2018, 2024; McNeely et al., 2020; Na et al., 2015, 2018). Recently, the *Drosophila* model system has emerged as a promising model for human nephrolithiasis research (Wang et al., 2022). A recent study using *Drosophila* successfully generated calcium oxalate-based stones within the MT and confirmed the role of the oxalate co-transporter (SLC26A6) and excess zinc in stone formation (Chi et al., 2015). Renal stones are a high-risk factor for renal-related disease, and their prevalence is increasing along with that of various diseases in humans. However, research on the physiological mechanisms of renal stones, drug discovery, aging, and stress, is insufficient. Research on the relationship between the back and renal stone formation is limited.

Here, we aimed to determine the association between increased CA in renal stem cells and kidney stone formation due to aging and oxidative stress in a *Drosophila* kidney model. Our data provide the first direct evidence of CA in RSCs and increased renal stone formation with age and oxidative stress. In addition, high sucrose feeding showed increased stone formation. These results show that renal cancer and stone formation are closely related to aging, ROS stress, and chronic diseases, such as diabetes and obesity. Furthermore, we argue that the *Drosophila* model system is suitable for studying emerging renal stone models, and that it may provide a platform for drug discovery.

## RESULTS

### Age and oxidative stress-induced increase of CA in mitotic RSC

**Fig. 1.**
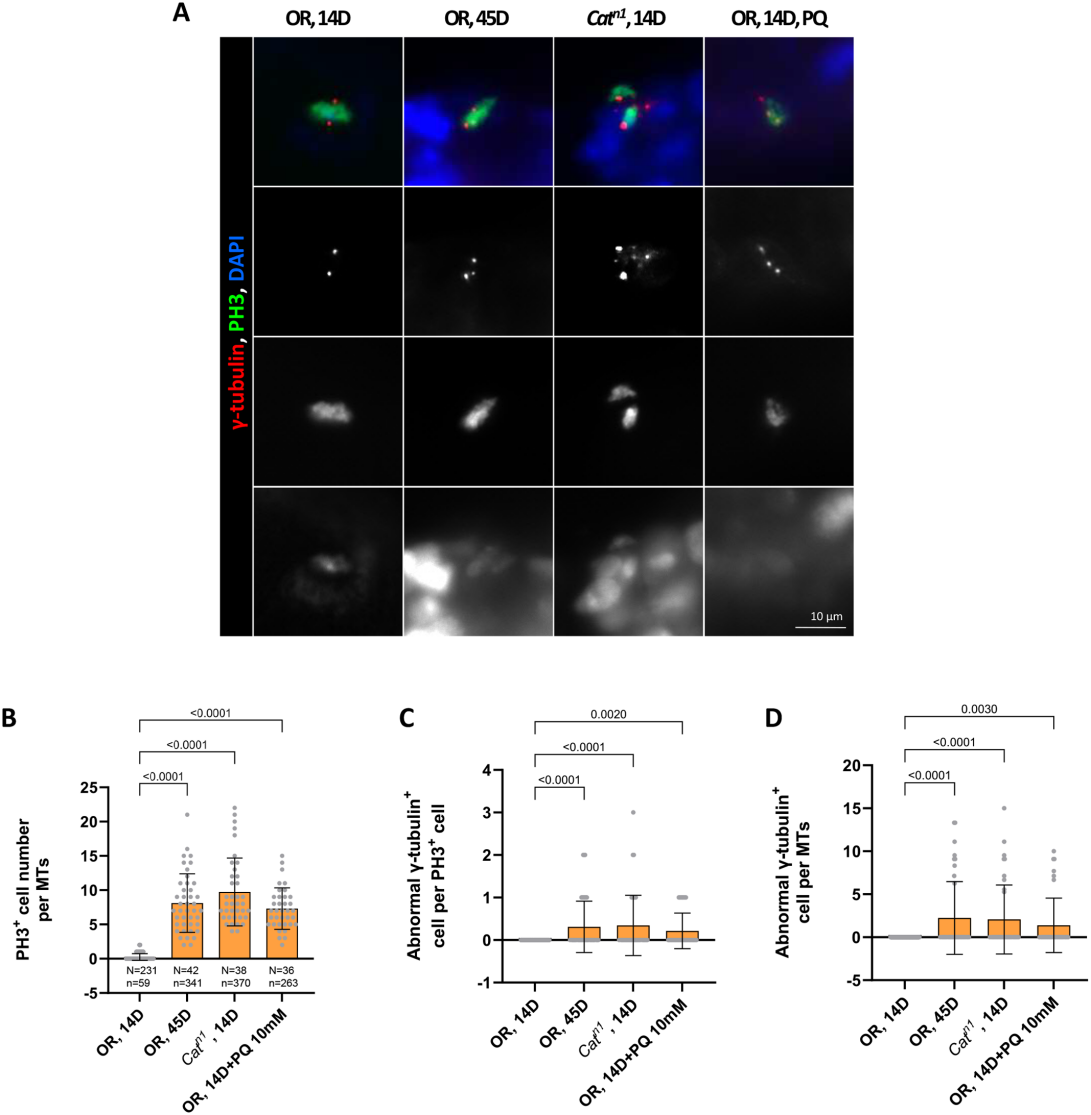
**CA in mitotic RSCs of aged and oxidative stressed *Drosophila* MT.** (A) Representative images of γ-tubulin signal in mitotic RSCs (PH3^+^ cells) of 14- and 45-day-old wild-type, 14-day-old *Cat^n1^*/*+*, and PQ-treated 14-day-old wild-type flies. The whole guts with MT of 10-day-old wild-type flies were stained with anti-PH3 (green) and anti-γ-tubulin (red). Original magnification is 400×. (B-D) Increased number of mitotic RSCs with supernumerary centrosomes in the MT of 14- and 45-day-old wild-type flies, 14-day-old *Cat^n1^*/*+*, and PQ-treated 14-day-old wild-type flies. (B) Age- and oxidative stress-related increases of mitotic RSCs in the whole guts with MT. (C) Frequency of abnormal γ-tubulin cell per mitotic RSC. (D) Number of abnormal γ-tubulin cell per the MT. The whole guts with MT of 14- and 45-day-old wild-type flies, 14-day-old *Cat^n1^*/*+*, and PQ-treated 14-day-old wild-type flies were stained with anti-PH3 (green) and anti- γ-tubulin (red). The centrosome numbers were counted in the PH3^+^ cells of these MTs. Data (mean±s.e.) in 14- and 45-day-old wild-type flies, 14-day-old *Cat^n1^*/*+*, and PQ-treated 14-day-old wild-type flies were collated from 59, 341, 370, and 263 mitotic cells of 231, 42, 38, and 36 flies, respectively, from three separate experiments. Data are presented as the mean±s.d. *P*-values compared to that of 14-day-old wild-type flies.

Using anti-γ-tubulin (a centrosome marker) and anti-PH3 (a mitotic RSC marker), we investigated whether centrosome duplication in mitotic RSCs is modulated by aging in the adult MT. Whole guts with MT of 14- and 45-day-old wild type (OR) were examined, and 14-day-old *Catalase* mutant (*Cat^n1^*/*+*) and PQ-treated 14-day-old wild-type flies were used as models of oxidative stress on tissues. Generally, two centrosomes were detected in RSCs in the mitotic phase of 14-day-old flies, but mitotic intestinal stem cells with to 3-7 abnormal centrosomes were detected in aged *Drosophila* MT (Fig. 1A). We quantified the frequencies of these mitotic RSCs with supernumerary centrosomes, which were 2.25% in 45-day-old wild-type flies (*N*=42, *n*=341, where *N* indicates the number of whole guts with MT and *n* indicates the number of PH3^+^ cells), 2.06% in 14-day-old *Catalase* mutants (*N*=38, *n*=370), 1.38% in PQ-treated 14-day-old wild-type flies (*N*=36, *n*=263), and 0% in 14-day-old wild-type flies (*N*=231, *n*=59) (Fig. 1B-D). The number of mitotic RSCs with supernumerary centrosomes per MT was 0.31 in 45-day-old wild-type flies, 0.34 in 14-day-old *Catalase* mutants, 0.17 in PQ-treated 14-day-old wild-type flies, and 0 in 14-day-old wild-type flies (Fig. 1D). Overall, these results indicate a higher incidence of CA in aged and oxidative stressed RSC populations. These results suggest that CA is a hallmark of cancer due to aging and oxidative stress in the *Drosophila* renal model system.

### Age and oxidative stress-induced increase of renal stone formation in MTs

**Fig. 2.**
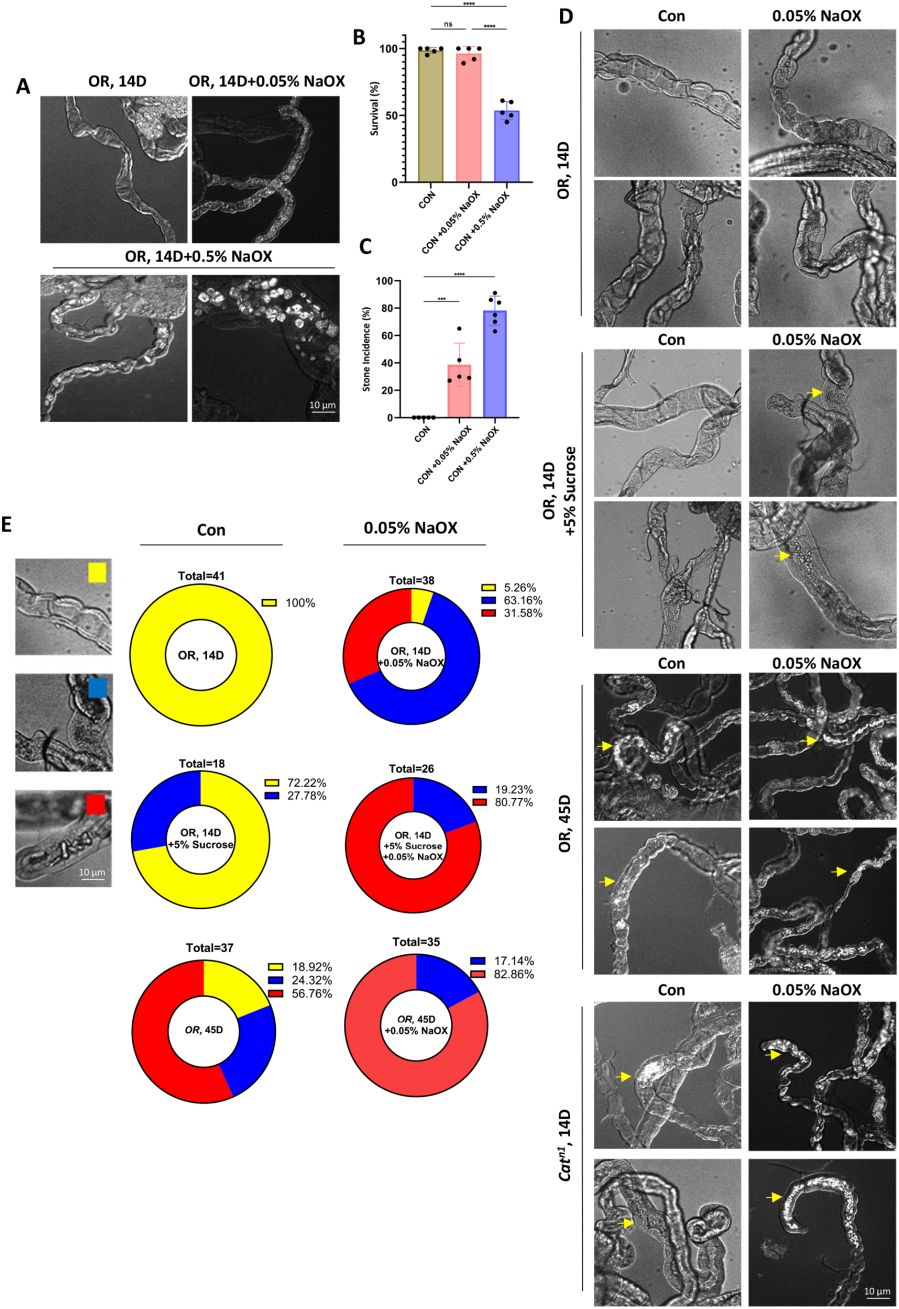
**Renal stone in age-, oxidative-stress-induced, and high sucrose in *Drosophila* MT.** (A) Image of control, 0.5% NaOX, and 0.05% NaOX for 4 days fed OR flies MTs. (B) Survival of control, 0.5% NaOX, and 0.05% NaOX for 4 days fed flies. (C) Stone incidence (%) control, 0.5% NaOX, and 0.05% NaOX for 4 days fed flies. (D) MTs Image of 14-day-old OR flies, 14-day-old OR flies with 0.05% NaOX, 14-day-old OR flies with 5% sucrose, 14-day-old OR flies with 5% sucrose mixed 0.05% NaOX, 45-day-old OR flies, 45-day-old OR flies with 0.05% NaOX, 14-day-old *Cat^n1^*/*+* flies, and 14-day-old *Cat^n1^*/*+* flies with 0.05% NaOX were fed for 7 days. Yellow arrow indicated impurities or stone formation in *Drosophila* MT. (E) Quantification graph of stone formation rate (%) in MTs of 14-day-old OR flies, 14-day-old OR flies with 0.05% NaOX, 14-day-old OR flies with 5% sucrose, 14-day-old OR flies with 5% sucrose mixed 0.05% NaOX, 45-day-old OR flies, 45-day-old OR flies with 0.05% NaOX, 14-day-old *Cat^n1^*/*+* flies, and 14-day-old *Cat^n1^*/*+* flies with 0.05% NaOX. Color indicated none (yellow), impurities (blue), and stone formation (red). Data are presented as the mean±s.d. ns., non-significant, ***P<0.001. ****P<0.0001.

In general, the incidence of kidney stones is known to increase due to a variety of factors such as genetics, diet, medications, and hyperoxaluria, which contribute to the onset of calcium oxalate (CaOx) stone development (Ali et al., 2018; Boyd et al., 2018). *Drosophila* MT regulates the levels of calcium, magnesium, and oxalate in the organ, and these ions in particular directly affect stone formation (Wang et al., 2022). Building on previous findings, we sought to determine whether stone formation could be induced in *Drosophila* by using a stone-inducing agent. Our experiments revealed that treatment with sodium oxalate (NaOX), a known inducer of calcium stones, resulted in stone formation in a concentration-dependent manner. Interestingly, as the stone formation rate increased, fruit fly survival decreased (Fig. 2A,B). This suggests that excessive stone formation in humans may also negatively affect lifespan. Another research group has established several new models of kidney stones using dietary and genetic approaches (Wang et al., 2022). For example, adult *Drosophila* supplemented with NaOX and ethylene glycol form CaOx crystals separately (Wang et al., 2022). Specifically, NaOX administration supplies dietary oxalate that combines with Ca^2+^ to form CaOx stones in MT (Chou et al., 2011; Wang et al., 2022; Cohen et al., 2020). These results suggest that the *Drosophila* model is a promising platform for studying renal stone disease in humans.

Based on the results shown in Fig 1 and Fig 2A,B, we sought to confirm the correlation between the rate of stone formation, aging, and oxidative stress. We analyzed the results by specifying three different groups: none (yellow), impurities (blue), and stone formation (red) in *Drosophila* MT. No stone formation (yellow) was observed in normal young *Drosophila* MT (Fig. 2D,E). When fed with 0.05% NaOX for 7 days, impurities were mixed (63%, blue), small stones were formed (31%, red), and stones that were not yet formed (5%, yellow) were mixed in each *Drosophila* MTs (Fig. 2D,E). When 5% sucrose was added for 7 days, normal (72%, yellow) and impurities (28%, blue) appeared (Fig. 2D,E). Under these conditions, when 0.05% NaOX was included in the 7-day treatment, the rate of stone formation increased to 80% (red), impurities increased to 20% (blue), and no normal MT was observed compared to the control group (Fig. 2D,E). It is particularly interesting that in aged MTs, the proportion of MTs with at least one stone was over 56% (red), even though they were not treated with stone-inducing agents, compared with young flies (Fig. 2D,E). When stone-inducing agents were used with aging, the rate of stones increased to 82% (red), and the MT group became full of stones compared to the non-treatment group (Fig. 2D,E). In addition, in the MTs of the *Cat^n1^* mutant line with increased oxidative stress, the proportion of MTs with at least one stone was more than 30% (red), even though they were not treated with a stone-inducing agent, compared to young flies (Fig. 2D,E). When the *Cat^n1^* mutant line was treated with a stone-inducing agent, the rate of stone formation increased to 90% (red), and the MTs became full of stones, compared with the control groups (Fig. 2D,E). Our results indicate that renal stones were induced by age, oxidative stress, and high sucrose levels in *Drosophila* MTs.

## DISCUSSION

In this study, we found that the first direct evidence of CA in RSCs and increased renal stone formation with age and oxidative stress. In addition, it was also shown for the first time that high sucrose feeding increased renal stone formation.

Previously, we reported that age-related increase of abnormal centrosome caused by DNA damage accumulation in *Drosophila* ISCs (Park et al., 2014, 2018). Here, we also checked DNA damage accumulation in MT, γH2AX increased with age and oxidative stressed condition (data not shown). DNA damage in the kidney has been shown to contribute to both acute and chronic renal injury, as well as to renal cell carcinoma (Garaycoechea et al., 2023). Analysis of DNA damage-based cancer hallmark in *Drosophila* renal system will provide essential basic information of nephrotoxicity when exposed to various condition, including those involving aging, ROS, and cancer therapies.

Other groups identified several risk factors for renal stones, including sex, age, high sodium intake, hypercalcemia, metabolic abnormalities, and obesity (Abufaraj et al., 2021; Ye et al., 2023). Notably, other studies have indicated that an unhealthy metabolic status significantly contributes to the onset and recurrence of renal stones (Lee et al., 2016; Boyd et al., 2018). In addition, oxidative stress caused by ROS is a major risk factor for calcium oxalate nephrolithiasis. Reports suggest that the oxidative stress response is induced in mammals and humans with renal stones (Ushimoto et al., 2023). The risk of developing kidney stones increases with age, affecting approximately 10% of people over their lifetime (Wang et al., 2020). Our findings support the notion that stone formation in the kidneys may increase due to aging or oxidative stress. In addition, under these conditions, the severity of stone formation due to stone-inducing agents increases significantly. This implies that individuals may be more susceptible to other environmental factors, dietary habits, and renal diseases. These results demonstrate a close relationship between kidney stones and chronic conditions, such as aging, ROS stress, diabetes, and obesity. We propose that the *Drosophila* kidney stone model is an effective tool for studying the relationship between stone formation and renal disease.

The *Drosophila* model can also be instrumental in the discovery and analysis of new agents for the control of age-related kidney diseases, including synthetic compounds, natural compounds from medicinal plants.

## MATERIALS AND METHODS

### *Drosophila* stocks, culture, and husbandry

Stocks were maintained on standard food media under a ∼12 h:12 h light:dark cycle at 25°C. The standard food consisted of 15.8 g yeast, 9 g soy flour, 5.2 g agar, 67 g cornmeal, and 0.5% propionic acid. To avoid larval overpopulation, <30 adult flies per vial were transferred to new standard food vials every 2-3 days. *Oregon-R* represents wild-type flies. The model of intrinsic oxidative stress, *Catalase* heterozygous mutant flies (*Cat^n1^* mutant flies), was provided from Bloomington *Drosophila* Stock Center (Bloomington, IN, USA).

### Paraquat (PQ) feeding assay

For the PQ feeding assay, OR flies were fed 10 mM PQ (working) in standard food media for 18-20 h at 25°C, and the guts were then analyzed via immunostaining.

### NaOX feeding assay

Five-day-old and 45-day-old OR flies, as well as 5-day-old catalase mutant flies were fed 0.5% or 0.05% NaOX (Sigma-Aldrich, St. Louis, MO, USA) mixed in standard food for 7 days at 25°C. The high dietary sugar feed method was employed, as previously described (May et al., 2019). Five-day-old OR flies were fed 5% sucrose or 0.05% NaOX and 5% sucrose in standard media for 7 days at 25°C. After feeding, the entire gut containing MT was dissected and analyzed. The flies were transferred to new food vials every 2-3 days.

### Immunochemistry for fly MT

For immunostaining, the whole intact gut with MT was dissected. Thereafter, the whole gut was fixed with MT for 1 h in 4% formaldehyde (Sigma-Aldrich) at 25°C. For co-immunostaining with primary antibodies, the whole gut with MT was fixed for 30 min in 4% paraformaldehyde in 1× phosphate buffered saline (PBS) (Electron Microscopy Science, Hatfield, PA, USA). The samples were dehydrated with methanol and rehydrated for 5 min in 50 and 25% methanol in PBST (0.1% Triton X-100 in 1× PBS). After washing thrice for 20 min with 1× PBST, the samples were incubated overnight at 4°C. After washing thrice for 20 min with 1× PBST, the samples were incubated at 25°C for 1 h with the secondary antibodies and DAPI (4′,6-diamidino-2-phenylindole; 1;1000; Molecular Probes, Eugene, OR, USA), and washed again thrice for 20 min in 1× PBST. Subsequently, samples were mounted using Vectashield (Vector Laboratories, Burlingame, CA, USA) and analyzed using an Axioskop 2 Plus microscope (Carl Zeiss Inc., Göttingen, Germany). The number of phospho-histone H3^+^ (PH3^+^) cells was counted in the whole MT.

### Antisera

The following primary antibodies were used in this study: rabbit anti-PH3 (Cat# 06-570, RRID:AB_310177, Millipore, Billerica, MA, USA), 1:1000; mouse anti-γ-tubulin (Cat# T6557, RRID:AB_477584, Sigma-Aldrich), 1:1000. The following secondary antibodies diluted in 1× PBST were used in this study: goat anti-rabbit FITC and goat anti-mouse Cy3 (Cat# 111-095-003, RRID: AB_2337972, Cat# 115-165-003, RRID: AB_2338680, Jackson ImmunoResearch, West Grove, PA, USA), 1:400.

### Quantitative analysis

The number of PH3 positive cells in the entire MT was counted for quantitative analysis. To quantify CA, the number of γ-tubulin-stained spots per PH3 positive cell in the whole MT was determined. The quantified data are expressed as means±s.e. Significant differences were determined using Student's *t*-test. Sigma Plot 14.5 (Systat Software Inc., San Jose, CA, USA) was used to analyze standard error.

### Statistical analysis

Data representation and statistical analyses were performed using GraphPad Prism software. Statistical analysis was performed using a *t*-test, and multiple comparisons were performed using one-way ANOVA. All experiments were independently replicated three times.
